# Is Obesity a Risk Factor for Wheezing Among Adolescents? A Prospective Study in Southern Brazil

**DOI:** 10.1016/j.jadohealth.2012.08.016

**Published:** 2012-12

**Authors:** Ricardo B. Noal, Ana M.B. Menezes, Silvia E.C. Macedo, Samuel C. Dumith, Rogelio Perez-Padilla, Cora L.P. Araújo, Pedro C. Hallal

**Affiliations:** a Epidemiology Postgraduate Program, Federal University of Pelotas, Pelotas, Brazil; b Instituto Nacional de Enfermidades Respiratorias, Mexico, D.F., Mexico

**Keywords:** Body mass index, Obesity, Skinfold, Wheezing, Adolescence, Longitudinal

## Abstract

**Purpose:**

To investigate the effect of obesity at the start of adolescence on the prevalence, incidence and maintenance of chest wheezing among individuals aged 11–15 years in a birth cohort in a developing country.

**Methods:**

The seventh follow-up of the 1993 Pelotas birth cohort occurred in 2004 (individuals aged 10–11 years). Between January and August 2008, the eighth follow-up of the cohort was conducted. All the individuals of the original cohort who were alive (who were then adolescents aged between 14 and 15 years) were targets for the study. The International Study of Asthma and Allergies in Childhood (ISAAC) questionnaire was used to define wheezing. In addition to the body mass index (BMI), used to define obesity by the World Health Organization (WHO) criteria, we assessed skinfold thickness.

**Results:**

From the original cohort, 4,349 individuals were located (85.7% follow-up rate). The prevalence of chest wheezing at 11 and 15 years were 13.5% (95% CI: 12.5%–14.5%) and 12.1% (95% CI: 11.1%–13.1%), respectively. The prevalence of wheezing at both times was 4.5% (95% CI: 3.9%–5.1%) and the incidence of wheezing was 7.5% (95% CI: 6.7%–8.3%). Independent of the effect of various confounding variables, the prevalence of wheezing at 15 years was 50% greater among obese individuals than among eutrophic individuals at 11 years (RR 1.53; 95% CI: 1.14–2.05). The greater the skinfold tertile at 11 years, the higher the prevalence of wheezing at 15 years was (*p* = .011). Weight status and skinfolds did not present any association with incident wheezing. After controlling for confounding factors, the risk of persistent wheezing among obese individuals at 11 years was 1.82 (95% CI: 1.30–2.54).

**Conclusions:**

Since obesity at the start of adolescence is associated with asthma symptom persistence, prevention and treatment of obesity may reduce avoidable healthcare costs and disease burden.

**Implications and Contribution**

The prevalence of wheezing in the last 12 months at 11 and 15 years old was 4.5%. The incidence of wheezing from 11 to 15 years old was 7.5%. Obesity was associated with a higher risk of wheezing in this birth cohort. Prevention and treatment of obesity in adolescence may reduce respiratory morbidity at later ages.

The incidence and prevalence of asthma symptoms remain high throughout the Western world [1]. It is estimated that the prevalence of asthma in adolescents aged 13–14 years is 18.2% in the southern region of Brazil [2]. Moreover, because of the various morbid conditions associated with obesity, the increased body mass index (BMI) observed among the young population is a matter of concern [3–5]. The effects of obesity on respiratory symptoms, illustrated long ago by Charles Dickens in “The adventures of Mr. Pickwick,” have been studied for around half a century. Over recent decades, associations between obesity and asthma have begun to arouse greater discussion in the scientific media [6–8].

Today, obesity is described in the literature as one of the risk factors for asthma development, together with sex and genetic predisposition [9]. However, since a large proportion of the knowledge about this association comes from studies with a cross-sectional design, one of the most important criteria of causality, i.e., temporality, is not taken into consideration [10–16]. Systematic reviews and meta-analyses on the few longitudinal studies (which were all carried out in developed countries) have found that the risk of asthma development is up to 50% greater among obese individuals than among individuals who are considered to be eutrophic [17–19]. From assumptions that the prevalence of obesity among adult Americans was around 30% and that the risk of asthma occurrence among obese individuals was between 1.6 and 3.0. Ford et al. estimated that obesity could be responsible for around 15% to 38% of asthma cases [20].

The high occurrence rates of obesity and asthma in this age group, the lack of longitudinal studies conducted in developing countries, and the potentially modifiable nature of risk factors for asthma symptoms attributed to obesity were the justifications for carrying out the present study. In this light, the aims of this study were to investigate the effects of obesity at the start of adolescence on the incidence, maintenance and prevalence of chest wheezing at the age of 15 years among the 1993 birth cohort of the city of Pelotas.

## Method

### The 1993 Pelotas (Brazil) Birth Cohort Study

In 1993, all births that occurred in all hospitals in Pelotas—a city of approximately 340,000 inhabitants in the southern region of Brazil—were identified. The mothers answered a standardized questionnaire that sought information relating to the gestational period, and the newborns were weighed immediately after delivery. Since then, this population has been followed up periodically; details of the methodology used are available in other published papers [21–23].

### The follow-up

The seventh follow-up occurred in 2004 (individuals aged 10–11 years). Between January and August 2008, the eighth follow-up of the cohort was conducted. All the individuals of the original cohort who were alive (who were then adolescents aged between 14 and 15 years) were targets for the study. During the fieldwork, two different teams with periodic training and standardization were used. One team was responsible for interviews conducted in homes and the other, for anthropometric evaluations made at a measurement center; only adolescents who refused to go to the center underwent anthropometric evaluations at home.

### The outcomes

The questionnaire of the “International Study of Asthma and Allergies in Childhood Steering Committee” (ISAAC), which had already been validated in Brazil, was used to assess the outcome [24,25]. Through the questions “Since <MONTH> of last year has <NAME> had chest wheezing?”, which had been applied to the mother in 2004, and “Since <MONTH> of last year have you had chest wheezing?”, which was applied to the adolescent in 2008, the prevalence of chest wheezing at the ages of 11 and 15 years, respectively, were determined. With the aim of evaluating the trajectory of wheezing over the follow-up periods, three main outcomes were defined: prevalence of wheezing at 15 years of age; incident wheezing (individuals whose mothers had not reported any presence of wheezing at the age of 11 years but who themselves reported at the follow-up at the age of 15 years that wheezing was occurring); and persistent wheezing (those who presented chest wheezing at both follow-ups, i.e., who continued to present wheezing). The reference categories for analyzing incident wheezing and persistent wheezing were adolescents who did not present wheezing at the ages of 11 years and 15 years, and adolescents who presented wheezing at 11 years of age but not at 15 years of age, respectively.

### The anthropometric variables

The anthropometric variables of the study were defined through the means from two measurements of weight (using a Tanita digital balance with accuracy of 100 grams), height (using an aluminum stadiometer), and the triceps and subscapular skinfolds (Cescorf skinfold caliper, with accuracy of one millimeter), which were made during the follow-ups in 2004 and 2008 [26]. The BMI was then calculated in accordance with the reference values of the World Health Organization (WHO) for children and adolescents of school age [27]. These criteria classify individuals between the ages of 5 and 19 years according to the BMI Z-scores for age and sex. Because of the small number of individuals with Z-scores less than −1, the category “thin” was not used. Thus, individuals with Z-scores of up to +1 standard deviation (SD) were considered to be “normal”; those with Z-scores greater than one and up to +2 SDs were considered to present “overweight”; and those with Z-scores higher than +2 SDs were considered to be “obese.”

### The trajectory variables

With the aim of evaluating the trajectory of obesity from 11 to 15 years of age, the variable “trajectory of obesity” was created. This was categorized as never obese (no obesity either at 11 or at 15 years of age); obese at 11 and nonobese at 15 years; nonobese at 11 years and obese at 15 years; and always obese, i.e., at both follow-up times. Through summing the triceps and subscapular skinfold values (using the means from three measurements), the variable “skinfold sum at 11 years of age” was generated, and this was subsequently categorized into tertiles. The trajectory of the skinfolds at the two follow-ups was evaluated in five categories: maintenance in the first tertile; reduction of tertile; maintenance in the middle tertile; increase of tertile; and maintenance in the upper tertile.

### The confounding factors

To control for possible confounding factors, demographic characteristics (sex and self-reported skin color) and data relating to the mother's pregnancy and puerperal period that had been gathered in 1993 (maternal smoking during pregnancy, mother's age at delivery and newborn's gestational age) were used. Reasons for hospital admission during the first year of life were used as controls. The socioeconomic variable was based on changes in family income (described in terms of numbers of minimum salaries) between birth and 15 years of age and consisted of the following categories: always poor; not poor to poor; poor to not poor; never poor. At the follow-up in 2004, the following were assessed: household density, i.e., the number of people who slept in the same bedroom as the adolescent in question; the parents' smoking habit; and any family history of asthma or chest wheezing or bronchitis.

At the follow-up in 2008, the following were included as possible confounding factors: number of symptoms of gastroesophageal reflux disease (GERD)[28] seen per week and, through a confidential questionnaire, sexual maturation according to the Tanner stages (categorized sum of points relating to development of pubic hair and penis/breasts) [29,30].

From the information obtained at the follow-ups in 2004 and 2008, the trajectory of the adolescent's variables of smoking and physical activity, defined threshold of at least 300 minutes per week, was ascertained (not present either in 2004 or in 2008; present only in 2004; present only in 2008; present both in 2004 and 2008) [31]. The trajectory of medical diagnoses of allergy was also ascertained (without any medical diagnosis of rhinitis or eczema at either follow-up; rhinitis or eczema at one of the follow-ups; rhinitis and eczema at both follow-ups). In addition to the above variables, breastfeeding—during the first year of life, exclusive breastfeeding, and predominant breastfeeding—(1993–94), contact with cats or dogs (1997–98), age at menarche (2008) and use of medications over the two weeks preceding the interview (2008) were also evaluated as possible confounders.

### The statistical analysis

The data were double-entered into the Epi-Info 6.0 software and were analyzed using the Stata 9.0 statistical package (StataCorp, College Station, TX). Bivariate analysis, with calculation of proportions and 95% confidence intervals (95% CI), was performed using the chi-square test for heterogeneity or linear trend. Multivariable analysis to control for possible confounding factors was performed by means of Poisson regression, with robust adjustment for variance. The results were presented as prevalence or incidence ratios and the 95% CI. Since the effect of the exposures analyzed did not differ according to sex or socioeconomic level (*p* for interaction >.10), it was not necessary to stratify the analyses according to these variables. The significance level for the two-tailed tests was taken to be 5%.

### Ethical aspects

The parents or guardians signed an informed consent statement and assurances were provided regarding data confidentiality. The study was approved by the Research Ethics Committee of the Federal University of Pelotas, which is affiliated with the Brazilian National Research Ethics Council (CONEP).

## Results

### The 1993 Pelotas (Brazil) Birth Cohort Study

Out of the 5,249 members of the original cohort, 4,201 individuals were located (excluding 148 deaths), which corresponded to a follow-up rate at 15 years of age of 85.7%. Table 1 describes the distribution of the variables used for controlling for confounding factors in relation to the original cohort population and the population located at the age of 15 years. The difference in the distribution of the variables between the original cohort and cohort followed up was less than one percentage point. Among the individuals accompanied at the age of 15 years, just over half were female; most were white-skinned; one fifth had always been poor; approximately 60% were inactive at the age of 15 years; almost half of them said that they had had a medical diagnosis of allergies; and just over 80% said that they had never smoked.

### Weight status and wheezing—crude analysis

At the age of 11 years, approximately one third of the adolescents in the cohort had presented overweight or obesity (Table 2 ). At the next follow-up, less than 3% of the adolescents had become obese, while just over 6% remained obese. Almost one quarter of the adolescents remained in the upper tertile of the skinfold sum. The prevalence of chest wheezing at 11 and 15 years of age were 13.5% (95% CI: 12.5%–14.5%) and 12.1% (95% CI: 11.1%–13.1%), respectively. In relation to the adolescents who had not presented wheezing at 11 years of age, 8.7% (95% CI: 7.8%–9.6%) of the adolescents presented incident wheezing. In relation to the adolescents who had presented wheezing at 11 years of age, 33.4% (95% CI: 29.5%–37.3%) of the adolescents presented persistent wheezing. The presence of wheezing at the age of 15 years was greater among obese individuals than among nonobese individuals. Likewise, it was observed that the prevalence of wheezing at 15 years of age tended to be higher if the tertile of the skinfold sum presented at the age of 11 years had increased.

The weight status and skinfolds at 11 years of age, the trajectory of obesity and the trajectory of skinfolds from 11 to 15 years of age were not significantly associated with incident wheezing. Obese adolescents and those in the upper tertile of skinfolds presented greater occurrence of persistent wheezing. Similar to what was observed with the skinfold tertiles, persistent wheezing was present in almost one third of the eutrophic individuals and in half of the obese individuals at the start of adolescence (*p* = .009). Approximately half of the individuals who remained obese and just over 40% of those who remained in the upper tertile of skinfolds reported having persistent wheezing.

### Weight status and wheezing—adjusted analysis

Table 3 shows that the prevalence of wheezing at the age of 15 years was 50% greater among the obese individuals than among the eutrophic individuals at the age of 11 years (*p* = .004). The higher the skinfold tertile at 11 years of age, the greater the prevalence of wheezing at 15 years of age (*p* = .011). Both in the crude and in the adjusted analysis, weight status and skinfolds were not associated with incident wheezing. After controlling for confounding factors, the risk of persistent wheezing among obese individuals at the age of 11 years almost doubled. Moreover, it was observed that negative confounding occurred in most of the analyses, since the adjusted effect estimate was greater than the crude measurement. Individuals who remained obese presented a chance of persistent wheezing that was approximately 80% greater (independent of the effects of, for example, sex, smoking, histories of allergies, and sexual development) than what was seen among individuals who remained eutrophic (*p* = .011). Again, a similar effect was observed between the skinfold categories at 11 years of age and the trajectory of skinfolds.

The variables of breastfeeding, contact with cats or dogs, and age at menarche did not present significant associations with weight status and the outcomes under assessment. At the age of 15 years, 37% of the adolescents reported that they had used some type of medication within the last 15 days preceding the interview. Corticosteroid use was reported by only 41 adolescents (.9%) and did not affect the estimates.

## Discussion

In this birth cohort conducted in a developing country that followed up adolescents from 11 to 15 years of age, it was observed that obesity at the start of adolescence was associated with occurrences of wheezing at 11 and 15 years of age and with maintenance of wheezing from 11 to 15 years of age, independent of various mediating and confounding factors. No association between obesity at the start of adolescence and incident wheezing was observed.

These findings gain importance because they came from a population-based cohort that had been followed up at different times, with low numbers of losses over the 15 years, as demonstrated in Table 1. In the present study, the chance of residual confounding was low because not only were the main factors described in the literature as capable of mediating or confounding the association between obesity and wheezing taken into account, but also the confounding observed was basically negative. It should also be emphasized that, in accordance with guidance from WHO, quality control on BMI and skinfold measurements was performed through standardization and refreshment throughout the fieldwork, which enabled an adequate assessment of the presence of obesity.

On the other hand, we cannot fail to highlight the main limitation of this study, which related to the diagnosing of asthma. The definition of asthma put forward by the Global Initiative for Asthma (GINA), i.e., that this is a disease with the presence of chronic inflammation, hyperresponsiveness of the lower airways and airflow limitations that are reversible either spontaneously or in response to use of a bronchodilator, is unviable from an epidemiological point of view [32]. Because of this, the diagnosing of asthma (which has repeatedly been cited as the main limitation of studies) was done through medical diagnostic reports or through self-reported symptoms or use of medications for asthma. In order to identify individuals presenting wheezing, which is the main symptom of asthma, it was decided to apply the ISAAC questionnaire (which has been validated in several countries, including Brazil) because applying it in epidemiological studies is feasible and its findings can be compared with results already reported in the literature [24,33]. Because of the limitations related to diagnosing asthma, the term “chest wheezing” was used as an outcome in the present study, which corresponds to the description in the instrument used.

The presence of respiratory symptoms among obese individuals, even in the absence of airflow obstruction or hyperreactivity, may lead to errors in diagnosing asthma (misdiagnosis) in such populations [34]. In the present study, among the individuals who reported having received a medical diagnosis of asthma during the preceding year, the proportions of reports of chest wheezing were similar among normal, overweight, and obese individuals: 33%, 36%, and 37%, respectively (data not shown). Thus, weight status is not believed to have interfered with the association between wheezing and the medical diagnosis of asthma. This observation is in agreement with Aaron et al. [35], who suggested that with the exception of symptoms reported in visits to the emergency services, a situation in which the chance of misdiagnosing asthma is four times greater among obese individuals, overdiagnosing of asthma occurs similarly among obese and nonobese individuals [35,36]. Therefore, it is unlikely that the association between obesity and respiratory symptoms gave rise to any differential error regarding occurrences of chest wheezing.

Given that corticosteroid use is associated both with asthma and with obesity, it has been debated in the literature whether using this medication (which is preferred for treating persistent asthma) might not be a confounding factor [32]. In the present study, inclusion of the variable of corticosteroid use in the analysis model produced negative confounding, i.e., the measurements of the effect of obesity on occurrences of wheezing increased after adjustment for this variable. However, in agreement with another study conducted in Latin America, where the use of inhaled corticosteroids was low, less than 1% of the adolescents in this cohort reported having made use of this medication. As the proportion of inhaled corticosteroid use was similar between obese and nonobese patients, we decided to exclude this variable from the model [37].

Over the years, cross-sectional and case-control studies have consistently demonstrated, among a variety of populations and ages, especially in adults, that there is an association, often with dose-response effects, between increased BMI and occurrences of respiratory symptoms [20,38–40]. In our study, it was observed that obesity at the age of 11 years presented an association with wheezing at this age. However, obesity at the age of 15 years did not present an association with the prevalence of wheezing at the age of 15 years (data not show). These findings may suggest that obesity has greater influence during childhood and at the start of adolescence, regarding occurrences of asthma [41,42]. However, cross-sectional analyses have a limited capacity for affirming causality.

In a longitudinal study, Chinn and Rona observed that children who were obese at 5–6 years of age presented a risk of asthma at 10–11 years of age that was four times greater than shown by those with normal BMI at the start of the follow-up [42]. In the same paper, these authors emphasized the possibility that an epiphenomenon might have occurred, because certain risk factors were shared and because of the perception that the prevalence of these two conditions tended to increase in parallel [42]. Subsequently, the same authors further suggested that asthma might cause obesity through the use of medications, social stigma, and low physical capacity [43]. However, other longitudinal studies carried out in developed countries have suggested that obesity should be taken to be a risk factor for incident and persistent asthma [41,44,45].

**Figure 1:**
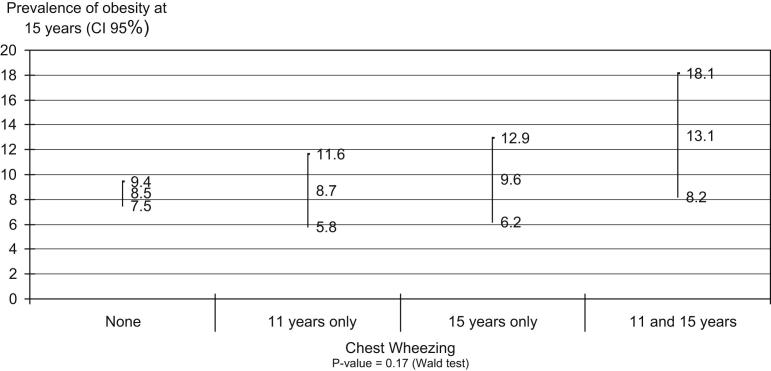
Prevalence of obesity at 15 years old (outcome) according to chest wheezing history (exposure). The 1993 Pelotas (Brazil) Birth Cohort Study.

In the present study, following up individuals from the start of adolescence to the age of 15 years made it possible to investigate certain facets of the association between obesity and wheezing. With the specific objective of investigating the possibility of bi-directionality, a stratified analysis on the trajectory of wheezing from 11 to 15 years of age was used to evaluate the risk of occurrence of obesity at 15 years of age (Figure 1 ). It was observed that since the risk of being obese did not differ between the categories of wheezing, occurrences of asthma at the start of adolescence did not seem to alter the natural history of obesity. This observation reinforces the direction of the association between obesity and asthma, thus suggesting that the causal chain during adolescence is unidirectional.

After the unidirectional nature of the relationship had been confirmed, the effect of obesity on wheezing was investigated. Longitudinal analysis showed that the risk of persistent wheezing (which was present in one third of the adolescents with wheezing at the age of 11 years) was 80% greater among obese individuals than among eutrophic individuals, after controlling for confounding factors. In agreement with the literature, this suggests that obesity is associated with maintenance and severity of asthma [44,46,47]. However, contrary to some studies [45,48], no association was observed between obesity at the start of adolescence and incident asthma. The time of life at which obesity has greatest impact on the risk of asthma development may be one of the key points in this question. In the cohort, the effect of obesity at 11 years of age on maintenance of wheezing occurred independently of obesity at the age of four years (data not shown) and at 15 years, confounding and mediating factors, respectively. In addition, after controlling for these factors, it was observed that individuals who were obese at the age of 11 years presented a risk of persistent wheezing that was almost three times greater (RR 2.8; 95% CI: 1.7–4.7) than among eutrophic individuals (data not shown). These data may suggest that the most sensitive time is the start of adolescence: a period that is characterized by intense hormonal and metabolic transformation. Some authors have investigated an association between early menarche and asthma symptoms, and the effect of obesity on this relationship [44,49]. In the present study, the association between obesity and persistent wheezing occurred independently of the degree of sexual maturity at the age of 11 years. In fact, the controls increased risk from 50% to 60% among the obese 11 year olds. On the other hand, these data do not rule out the possibility that the lack of any association may have been due to the need for a longer follow-up period for respiratory symptoms to develop.

The finding that obesity precedes asthma led us to investigate whether the effect observed might not have occurred because of some other confounding factor. Given that many conditions have been recognized as associated with developing and triggering asthma [9], it is understandable that lack of control for several of these might frequently be cited as limitations in studies on obesity and asthma. In the present study, controlling for potential confounding factors helped to clarify some of the doubts in the literature. It could be seen that negative confounding existed, independent of the combined effect of variables such as: sex, trajectory of family income, exposure to certain allergens, exposure to smoking, physical activity, histories of allergy, respiratory illnesses and reflux symptoms. This was shown by the fact that inclusion of these variables in the model caused an increase in the risks of wheezing associated with obesity. Inclusion of breastfeeding and contact with cats and dogs separately in the model also produced negative confounding (data not shown).

In practically all the analyses, there was consistency between the results found for the exposure variables (WHO weight status and skinfold tertile) and the wheezing variables. It was observed that in relation to the reference categories, not only the individuals who were obese or in the upper tertile of skinfolds at 11 years of age, but also those who remained obese or remained in the upper tertile of skinfolds between the follow-ups, presented a greater risk of persistent wheezing.

Several hypotheses have sought to explain the mechanisms through which obesity is associated with asthma. It has been suggested that the asthma phenotype might be affected by obesity through direct mechanical effects, increased immune response, genetic mechanisms, or hormonal influences [50,51]. However, because of the reduction in lung volume, lack of eosinophilic inflammatory response, and differences in therapeutic response, with resistance to corticosteroids and greater difficulty in achieving control, Beuther proposed that asthma in obese individuals should be considered to be a new phenotype [52]. Environmental factors such as physical activity, diet, and birth weight may be connected with obesity, and combinations of these factors with genetic susceptibility may cause increased risk of asthma [53–55]. However, more than being a risk factor in isolation, obesity may be a lifestyle marker, insofar as the environment within which obese individuals live (diet, lack of exercise, and lower physical capacity) may explain the presence and/or worsening of asthma symptoms [42,56]. To expand the approach towards the public health problem of asthma, Brisbon et al. proposed that the concept of the built environment should include not only the air quality but also personal attitudes and diet [57].

Worldwide, occurrence of obesity and asthma in adult populations has reached epidemic proportions, becoming a great concern for the scientific community [1,58]. Within this population, measures targeting weight loss have caused reduction in the occurrence of asthma symptoms and severity [59,60]. Considering that overweight or obese adolescents have a higher risk of obesity in adulthood in relation to nonobese, we must focus on preventive measures in the former population [61]. Therefore, particularly at early adolescence, obesity should be considered a modifiable risk factor that may contribute to reducing the occurrence and persistence of asthma.

## Figures and Tables

**Table 1:** Description of the variables studied and comparison with the original population from the 1993 Pelotas (Brazil) Birth Cohort Study

Characteristic	Original N (%)	Traced in 2008 N (%)
Sex	5,248	4,018
Male	2,606 (49.7)	1,966 (48.9)
Female	2,642 (50.3)	2,052 (51.1)
Skin color	4,420	4,001
White	2,953 (66.8)	2,646 (66.1)
Black or mixed	1,259 (28.5)	1,165 (29.1)
Other	208 (4.7)	190 (4.8)
Maternal smoking during pregnancy	5,249	4,018
No	3,497 (66.6)	2,683 (66.8)
Yes	1,752 (33.4)	1,335 (33.2)
Maternal age at delivery (years)	5,248	4,017
<20	915 (17.4)	692 (17.2)
20–34	3,756 (71.6)	2,884 (71.8)
≥35	577 (11.0)	441 (11.0)
Gestational age (weeks)	5,193	3,981
≥37	4,582 (88.2)	3,558 (89.4)
<37	611 (11.8)	423 (10.6)
Hospitalizations in the first year	5,249	4,018
No	4,297 (81.9)	3,323 (82.7)
Diarrhea, ARI, or bronchospasm	430 (8.2)	324 (8.1)
Another reason	522 (9.9)	371 (9.2)
Parents smoking in 2004	3,382	3,073
No one	785 (23.2)	714 (23.2)
At least one former smoker	932 (27.6)	862 (28.1)
At least one smoker	1,034 (30.6)	935 (30.4)
Both smoker	631 (18.6)	562 (18.3)
Family history of asthma, wheezing, or bronchitis	4,356	3,947
No	2,856 (65.6)	2,587 (65.5)
Yes	1,500 (34.4)	1,360 (34.5)
Number of people co-sleeping with adolescent	4,425	4,008
Nobody	1,175 (26.6)	1,054 (26.3)
One to three	2,868 (64.8)	2,617 (65.3)
Four or more	382 (8.6)	337 (8.4)
Number of days with GERD symptoms weekly	4,324	4,015
None	3,631 (84.0)	3,377 (84.1)
One day	460 (10.6)	428 (10.7)
Two or more days	233 (5.4)	210 (5.2)
Medical diagnosis of allergy (rhinitis and/or eczema)	4,214	3,976
No	1,956 (46.4)	1,831 (46.1)
Rhinitis or eczema	1,812 (43.0)	1,731 (43.6)
Rhinitis and eczema	446 (10.6)	413 (10.3)
Physical activity trajectory from 11 to 15 years old	4,092	3,880
Inactive in both periods	1,549 (37.9)	1,459 (37.6)
Active only at 11 years	871 (21.3)	830 (21.4)
Active only at 15 years	826 (20.1)	785 (20.2)
Active in both waves	846 (20.7)	806 (20.8)
Family income from birth to 15 years old	4,224	.922
Ever poor	876 (20.8)	809 (20.7)
Not poor–poor	540 (12.8)	503 (12.9)
Poor–not poor	941 (22.2)	874 (22.3)
Never poor	1,867 (44.2)	1,726 (44.1)
Tanner' pubertal stages (score)	3,442	3,250
2–4 (least developed)	191 (5.6)	176 (5.3)
5–7	1,691 (49.1)	1,597 (49.2)
8–10 (most developed)	1,560 (45.3)	1,477 (45.5)
Adolescent smoking trajectory at 11 and 15 years old	4,135	3,952
No–no	3,310 (80.1)	3,173 (80.3)
Yes–no	53 (1.2)	49 (1.2)
No–yes	675 (16.3)	635 (16.1)
Yes–yes	97 (2.4)	95 (2.4)

ARI = acute respiratory infection; GERD = gastroesophageal reflux disease.

**Table 2:** Prevalence of wheezing at 11 years old and at 15 years old, incident wheezing and persistent wheezing according with weight status and skinfolds. The 1993 Pelotas (Brazil) Birth Cohort Study

	N (%)	Prevalence of wheezing at 11 years (2004) (IC95%)	Prevalence of wheezing at 15 years (2008) (IC95%)	Incident wheezing 11–15 years (IC95%)	Persistent wheezing 11–15 years (IC95%)
Occurrences		13.5 (12.5; 14.5)	12.1 (11.1; 13.1)	8.7 (7.8; 9.6)	33.4 (29.5; 37.3)
Weight status at 11 years old	4,441	.003^a^	.042^a^	.826^a^	.009^a^
Normal	3,080 (69.3)	12.6 (11.4; 13.8)	11.6 (10.5; 12.8)	8.8 (7.7; 9.9)	30.4 (25.6; 30.1)
Overweight	888 (20.0)	13.9 (11.6; 16.2)	11.6 (9.4; 13.7)	7.9 (6.0; 9.9)	34.5 (25.8; 43.1)
Obese	473 (10.7)	17.8 (14.3; 21.3)	15.7 (12.4; 19.1)	9.1 (6.2; 12.0)	45.8 (34.8; 56.7)
Sum of skinfolds at 11 years old (tertile)	4,424	.207^a^	.053^a^	.611^a^	.023^a^
First	1,485 (33.6)	13.4 (11.7; 15,1)	11.0 (9.4; 12.6)	8.0 (6.5; 9.5)	29.7 (23.2; 32.2)
Second	1,465 (33.1)	11.9 (10.2; 13.6)	12.0 (10.3; 13.7)	9.6 (7.9; 11.3)	29.1 (22.1; 36.1)
Third	1,474 (33.3)	15.0 (13.2; 16.8)	13.4 (11.6; 15.2)	8.6 (7.0; 10.2)	40.2 (33.6; 46.8)
Obesity trajectory from 11 to 15 years old	4,032	.026^b^	.071^b^	.792^b^	.066^b^
No–No	3,498 (86.8)	13.2 (12.0; 14.3)	11.6 (10.5; 12.7)	8.6 (7.6; 9.6)	31.4 (27.1; 35.6)
Yes–No	181 (4.5)	19.9 (14.0; 25.8)	16.1 (10.7; 21.5)	9.7 (4.8; 14.6)	41.7 (24.8; 58.6)
No–Yes	102 (2.5)	12.7 (6.2; 19.3)	12.8 (6.2; 19.3)	11.2 (4.6; 17.9)	23.1 (-3.4; 49.6)
Yes–Yes	251 (6.2)	17.1 (12.4; 21.8)	15.9 (11.4; 20.5)	9.1 (5.2; 13.1)	48.8 (33.3; 64.4)
Skinfolds trajectory from 11 to 15 years old	4,030	.046^b^	.229^b^	.894^b^	.028^b^
Ever in the lowest tertile	923 (22.9)	13.8 (11.5; 16.0)	11.5 (9.4; 13.5)	8.2 (6.3; 10.1)	32.3 (24.0; 40.5)
Reduction of tertile	754 (18.7)	16.1 (13.5; 18.7)	13.0 (10.6; 15.4)	8.7 (6.5; 10.9)	33.9 (25.3; 42.4)
Ever in the middle tertile	650 (16.1)	11.5 (9.0; 13.9)	10.9 (8.5; 13.3)	8.2 (6.0; 10.5)	32.4 (21.5; 43.4)
Increase of tertile	769 (19.1)	11.9 (9.6; 14.2)	10.9 (8.7; 13.2)	9.5 (7.3; 11.7)	20.9 (12.4. 29.4)
Ever in the highest tertile	934 (23.3)	14.9 (12.6; 17.2)	13.9 (11.7; 16.1)	9.1 (7.1; 11.1)	41.7 (33.4; 50.0)

a Test for linear trend.

b Test for heterogeneity.

**Table 3:** Weight status and prevalence of wheezing at 15 years old, incident wheezing and persistent wheezing, crude and adjusted analyses. The 1993 Pelotas (Brazil) Birth Cohort Study

	Prevalence of wheezing at 15 years old		Incident wheezing from 11 to 15 years		Persistent wheezing at 11 and 15 years	
	Crude analysis	Adjusted analysis^a^	Crude analysis	Adjusted analysis^a^	Crude analysis	Adjusted analysis^a^
Weight status at 11 years old	.044^b^	.004^b^	.829^b^	.583^b^	.007^b^	.001^b^
Normal	1.00	1.00	1.00	1.00	1.00	1.00
Overweight	.99 (.81; 1.23)	1.19 (.92; 1.55)	.90 (.68; 1.18)	1.07 (.76; 1.51)	1.14 (.85; 1.52)	1.28 (.90; 1.83)
Obese	1.35 (1.07; 1.71)	1.53 (1.14; 2.05)	1.03 (.73; 1.45)	1.11 (.72; 1.71)	1.51 (1.14; 2.00)	1.82 (1.30; 2.54)
Sum of skinfolds at 11 years old (tertile)	.054^b^	.011^b^	.603^b^	.505^b^	.026^b^	.001^b^
First	1.00	1.00	1.00	1.00	1.00	1.00
Second	1.09 (.89; 1.34)	1.07 (.80; 1.43)	1.20 (.93; 1.55)	.94 (.65; 1.35)	.98 (.71; 1.35)	1.48 (.92; 2.37)
Third	1.22 (1.00; 1.48)	1.41 (1.07; 1.84)	1.07 (.82; 1.40)	1.12 (.78; 1.60)	1.35 (1.03; 1.78)	1.91 (1.26; 2.88)
Obesity trajectory from 11 to 15 years old	.065^c^	.083^c^	.788^c^	.398^c^	.039^c^	.011^c^
No–No	1.00	1.00	1.00	1.00	1.00	1.00
Yes–No	1.39 (.98; 1.96)	1.43 (.95; 2.17)	1.14 (.68; 1.89)	1.25 (.68; 2.31)	1.33 (.88; 2.00)	1.68 (1.00; 2.82)
No–Yes	1.10 (.66; 1.84)	1.39 (.74; 2.60)	1.31 (.73; 2.38)	1.71 (.88; 3.32)	.74 (.27; 2.00)	1.00 (.18; 5.46)
Yes–Yes	1.37 (1.02; 1.85)	1.44 (1.01; 2.07)	1.07 (.68; 1.66)	.99 (.56; 1.74)	1.56 (1.11; 2.18)	1.79 (1.20; 2.68)
Skinfolds trajectory from 11 to 15 years old	.229^c^	.049^c^	.894^c^	.781^c^	.039^c^	.003^c^
Ever in the lowest tertile	1.00	1.00	1.00	1.00	1.00	1.00
Reduction of tertile	1.13 (.88; 1.47)	1.05 (.74; 1.51)	1.07 (.76; 1.51)	.89 (.55; 1.44)	1.05 (.74; 1.50)	1.32 (.78; 2.23)
Ever in the middle tertile	.95 (.72; 1.26)	.97 (.65; 1.44)	1.01 (.70; 1.44)	.77 (.45; 1.29)	1.00 (.66; 1.52)	1.45 (.84; 2.53)
Increase of tertile	.95 (.73; 1.25)	.82 (.55; 1.23)	1.16 (.84; 1.61)	.94 (.58; 1.52)	.65 (.40; 1.04)	.49 (.22; 1.11)
Ever in the highest tertile	1.21 (.95; 1.54)	1.34 (.94; 1.92)	1.11 (.81; 1.53)	1.01 (.63; 1.64)	1.29 (.94; 1.78)	1.82 (1.10; 3.02)

a Adjusted for sex, skin color, maternal age at delivery, gestational age, hospitalization in infancy, maternal smoking during pregnancy, parents smoking at 11 and 15 years old, smoking trajectory of adolescent, income trajectory from birth to adolescence, family history of wheezing, medical diagnosis of allergy, physical activity trajectory from 11 to 15 years old, gastroesophageal reflux disease, Tanner's pubertal stage, and household density.

b Test for linear trend.

c Test for heterogeneity.
